# Experimental Investigation and CFD Modeling of Supercritical Adsorption Process

**DOI:** 10.3390/polym12091957

**Published:** 2020-08-29

**Authors:** Artem Lebedev, Daria Lovskaya, Natalia Menshutina

**Affiliations:** International Center for Transfer of Pharmaceutical and Biotechnology, Mendeleev University of Chemical Technology of Russia, Moscow 125047, Russia; daria.lovskaya@gmail.com (D.L.); chemcom@muctr.ru (N.M.)

**Keywords:** supercritical adsorption, active pharmaceutical substances, aerogels, CFD modeling

## Abstract

The kinetics of the supercritical adsorption process was experimentally studied by the example of ”ibuprofen-silica aerogel” composition obtainment at various parameters: Pressure 120–200 bar and temperature 40–60 °C. Computational Fluid Dynamics (CFD) model of the supercritical adsorption process in a high-pressure apparatus based on the provisions of continuum mechanics is proposed. Using supercritical adsorption process kinetics experimental data, the dependences of the effective diffusion coefficient of active substance in the aerogel, and the maximum amount of the adsorbed active substance into the aerogel on temperature and pressure are revealed. Adequacy of the proposed model is confirmed. The proposed mathematical model allows predicting the behavior of system (fields of velocity, temperature, pressure, composition, density, etc.) at each point of the studied medium. It makes possible to predict mass transport rate of the active substance inside the porous body depending on the geometry of the apparatus, structure of flow, temperature, and pressure.

## 1. Introduction

Today, one of the urgent tasks is the creation of new functional materials that can be used in various industries, for example, in pharmaceuticals, medicine, and biotechnology. Due to their unique properties, aerogels can be successfully used to create new materials by impregnating active pharmaceutical ingredients, cells, fluorescent materials, hydrogen sources, or gases into their pores [1,2,3,4]. Such materials can be used as drug delivery systems (creation of new drug compositions). As shown in some works [5], active substances adsorbed on the inner surface of an aerogel are predominantly in an amorphous state. Due to this, their release rate is significantly higher compared to the active substances in crystalline form. This leads to a corresponding increase in the bioavailability of drugs in aerogels [6], which leads to a faster onset of therapeutic effect.

The process of obtaining aerogel-based materials using supercritical fluid technology is called supercritical adsorption or supercritical impregnation. For successful impregnation into the aerogel, the active substance used must be soluble in the supercritical fluid. In some cases, a co-solvent [7,8] can be used to increase the solubility, which is selected depending on the nature of the active substance. The process of supercritical adsorption includes the dissolution of the active substance in the medium of supercritical fluid, the diffusion of the active substance into the aerogel together with the direct adsorption of its molecules on the inner surface of the pores of the aerogel, and the deposition of the active substance in the pores of the aerogel during depressurization [9]. The use of supercritical carbon dioxide as a supercritical fluid is preferable for the process of impregnation of active substances into the aerogel, since it is inert to many active substances and makes it possible to conduct the process at low temperatures (critical temperature of carbon dioxide is 31.1 °C). This is fundamentally important, since many active substances are not thermally stable. After removal of the supercritical fluid (under normal conditions, carbon dioxide is in a gaseous state), an aerogel with an impregnated active substance is obtained. One of the important characteristics of such a material is the maximum amount of the adsorbed active substance into the aerogel. The maximum amount reflects the maximum possible amount of the active substance at given process parameters, which is obtained when equilibrium is reached. The maximum amount depends on the external parameters of the supercritical adsorption process, the type of substance used, and the aerogel used, which is confirmed by studies of the impregnation of active substances into aerogels, which are presented in the literature [5,10,11,12,13,14]. Thus, it is clear that supercritical adsorption is a complex process that includes many phenomena and its study seems relevant.

In modern scientific and technical literature, the process of supercritical adsorption of substances into porous materials is described using the Langmuir, Dubinin–Astakhov theory, as well as the multicomponent potential adsorption theory [15,16,17]. However, these models describe processes in the local volume of the porous body, and not in the apparatus as a whole. In addition, these theories allow predicting the limiting values of adsorption and cannot be used to calculate the kinetics of the process. The application of CFD modeling methods allows taking into account the geometry of the apparatus and calculating the adsorption process in dynamics. It has been shown in many modern works that the CFD method can be successfully used to describe various supercritical processes: Supercritical extraction [18,19,20], micronization, and crystallization [21,22,23]. Moreover, using the indicated method, the hydrodynamics of the supercritical fluid inside the apparatus used can be calculated, as well as the heat and mass transfer of various components under arbitrary initial and boundary conditions. The use of such models allows a more detailed study of supercritical processes, in particular, their optimization and scaling.

The aim of present research is to study the process of supercritical adsorption in the preparation of the composition «aerogel-active substance». For this, the kinetics of the supercritical adsorption process and its dependence on the external parameters of the process were experimentally studied. A CFD model based on the provisions of continuum mechanics is also proposed. To select the model parameters and its validation, the experimental kinetics of supercritical adsorption are used. The developed model will allow to obtain a description of the supercritical adsorption process, depending on the parameters of the equipment and its structural characteristics.

## 2. Materials and Methods 

The following active substance is used in the present research: Racemic ibuprofen (RS-ibuprofen) is mixture of R-and S-enantiomers, which is a non-steroidal anti-inflammatory drug.

To obtain aerogels, tetraethoxysilane (TEOS) 98.5% (HimProm, Samara, Russia), isopropyl alcohol (C_3_H_8_O) 99.8% (RusHim, Moscow, Russia), food grade citric acid (C_6_H_8_O_7_) (RusHim, Moscow, Russia), and aqueous ammonia solution (NH_3_) (RusHim, Moscow, Russia) were used. These substances were used without additional purification, distilled water was obtained in the laboratory.

To carry out an experimental study of supercritical adsorption, silica aerogels in the form of cylindrical monoliths are used. To obtain silica aerogels was used method described in work [24]. Silica aerogel obtainment process consists of two main stages: Obtaining alcogels and their supercritical drying. Silica alcogels are obtained in accordance with a two-stage sol-gel process. The precursors are tetraethoxysilane and isopropyl alcohol, the catalyst at the stage of sol formation is an aqueous solution of citric acid, and the stage of gelation is an aqueous solution of ammonia. The reagents were taken in the following molar ratios: TEOS: C_3_H_8_O: H_2_O: C_6_H_8_O_7_: NH_3_ = 1.0 mol: 2.4 mol: 4.0 mol: 6.3 × 10^−3^ mol: 2.5 × 10^−2^ mol.

Supercritical drying of the obtained alcogels is carried out in an equipment for conducting supercritical processes [25]. The following process parameters are maintained: Temperature 40 °C, pressure from 120 to 140 bar, carbon dioxide flowrate 500–1000 g/h, drying time 4 h.

Analytical studies of the obtained aerogels were carried out by low-temperature N_2_ adsorption–desorption analysis using the equipment of the Center for Collective Use of the Mendeleev University of Chemical Technology of Russia. For this, the Micrometrics ASAP 2020(Micromeritics, Nocross, GA, USA) was used. The obtained adsorption and desorption curves were processed using the Brunauer–Emmett–Teller (BET) method to obtain the internal specific surface of aerogels and the Barrett–Joyner–Halenda (BJH) method to obtain the average diameter and pore volume. Prior to measurements, samples were dried under a vacuum at 60 °C for 20 h.

To accurately determine the amount of the adsorbed active substance into the aerogel, the method of high-performance liquid chromatography (HPLC) was used. The studies were carried out on an Agilent 1100 (Agilent Technologies, Santa Clara, CA, USA) equipped with a C-18 column, with a sorbent particle size of 3.5 μm and a column size of 4.5 by 75.0 mm (manufacturer Zorbax). Description of HPLC method: Mobile phase-water: acetonitrile: Orthophosphoric acid with ratio 660: 340: 0.5; flow rate 2.0 mL/min; column temperature 40 °C; and the light absorbance was read at 221 nm. For samples preparation a certain mass of aerogel suspensions with active substance were prepared. After incubation for one hour in a solution, the suspensions were centrifuged (10,000× *g*) for 10 min. The resulting solutions diluted 50 times, and then analyzed to obtain chromatographic curves. The area under the chromatographic curve (for certain sample) was recounted using the calibration curve in order to obtain concentration of the active drug in analyzed solution. This concentration (considering dilution) was converted into mass content of the active compound in the analyzed aerogel.

## 3. Experimental Investigation of Supercritical Adsorption Kinetics

**Figure 1:**
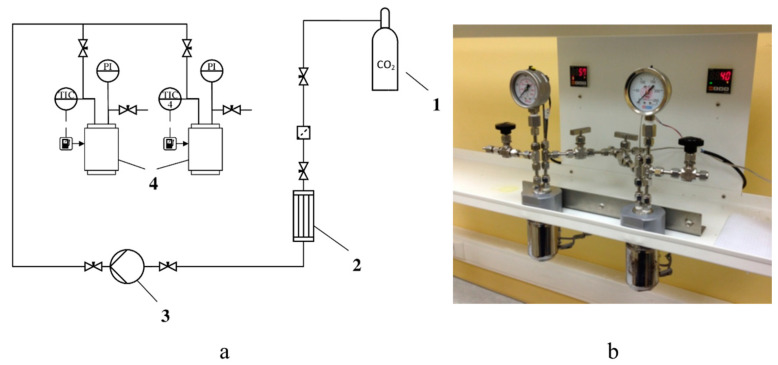
Scheme of the equipment for supercritical adsorption (**a**) and its outer look (**b**): 1–CO_2_ tank, 2–heat exchanger, 3–liquid high-pressure pump, 4–high pressure reactors with heating jacket, PI-manometers, and TIC-temperature controller with operator panel.

The kinetics of supercritical adsorption is studied using a special equipment for supercritical adsorption. Such an equipment was designed as part of this work. Scheme and its outer look are presented on the Figure 1.

Carbon dioxide is supplied using a Maximator high-pressure pump 1, which allows pump over liquid carbon dioxide. The suction line of the pump leads to CO_2_ tank, the discharge line leads to the high pressure reactors 4. The jacket of the reactor is able to maintain a temperature of not more than 200 °C. The voltage supplied to it is regulated by the proportional-integral-derivative controller (PID). The pressure in the reactors is monitored by manometers PI. The working volume of each reactor is 60 mL, the maximum working pressure is 250 bar.

To study the process of supercritical adsorption and determine the coefficients of the kinetics model, experimental studies of the kinetics of this process were performed. Experimental studies of the supercritical adsorption process consist in obtaining kinetics curves of this process. To obtain each point of this curve, a separate independent experiment is necessary, since in real time it is impossible to determine the change in the content of active substance in the aerogel.

Adsorption in the framework of this study was carried out into silica aerogel monoliths of a cylindrical shape with a diameter of 10 mm and a length of 50 mm. The parameters of the supercritical adsorption process varied: Pressure from 120 to 200 bar and temperature from 40 to 60 °C. To obtain each point of the kinetics curve, a certain mass of the active substance was placed in an envelope of filter paper. A silica aerogel monolith was loaded into the reactor for supercritical adsorption together with an envelope with active substance. The air from the apparatus was displaced by blowing carbon dioxide at atmospheric pressure low carbon dioxide flowrate after apparatus sealing. Then carbon dioxide was supplied to the reactor and the necessary process parameters were set. Pressurization and heating of the apparatus was carried out for no more than 10 min by simultaneously supplying carbon dioxide and increasing the power of the heating. The mass of the active substance was chosen so that its amount was in significant excess. The mass should be sufficient both to ensure the maximum concentration of the active substance (solubility) in the supercritical solution, and to its adsorption into aerogel. After keeping the apparatus under given conditions (temperatures 313, 323, and 333 K and pressure 120, 140, 160, and 200 bars) for a given time (1, 2, 3, 4, and 5 h for every given conditions) without any stirring, the pressure was released from the apparatus. Mean value of depressurization rate was about 10 bars per minute. It was fast enough at the beginning (within 1–3 min to a pressure of 70–80 bar) and slowly in the region of the critical pressure transition. The obtained samples were studied analytically to determine the amount of the adsorbed active substance into the aerogel using the HPLC method. Each point of the kinetics curve was repeated twice to determine reproducibility and standard deviation was calculated. The loading of the active substance at each point is the ratio of the mass of ibuprofen to the total mass of ibuprofen and aerogel.

## 4. Results and Discussion

The silica aerogels in the form of monoliths, obtained in accordance with the procedure, were studied by nitrogen porosimetry. The following characteristics of the indicated material were obtained: Internal specific surface area 975 m^2^/g, average pore diameter 4.1 nm, and pore volume 1.45 cm^3^/g. The silica aerogels obtained within the framework of the work have a developed internal structure and small pore size, which are typical for aerogels of this type.

**Figure 2:**
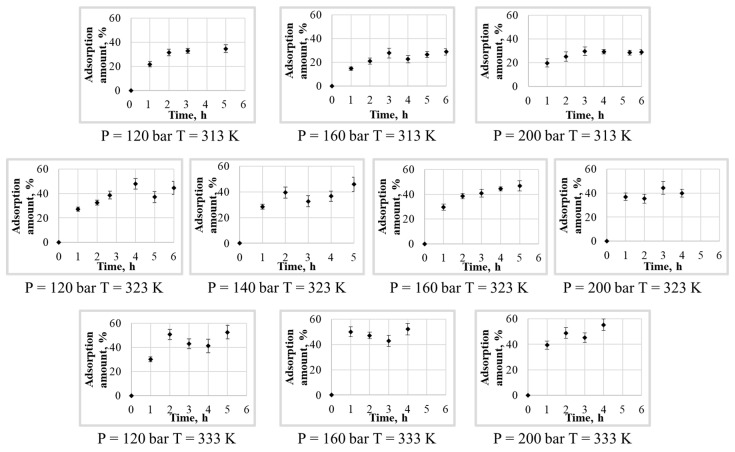
The results of an experimental study of the kinetics of supercritical adsorption.

As part of the study of the kinetics of the supercritical adsorption process, only ten series of experiments were performed on the adsorption of ibuprofen into silica aerogel at various pressures (P = 120, 140, 160, 200 bar) and temperatures (T = 313, 323, 333 K). The data thus obtained are presented in Figure 2 with standard deviation bars. The data are used in the work to find the necessary coefficients (maximum amount of the adsorbed active substance into the aerogel and effective diffusion coefficient relations on pressure and temperature) of the mathematical model and verify its adequacy.

As can be seen from the results obtained, the amount of the adsorbed active substance into the aerogel reaches its limit (or maximum) values on average over a period of not more than 5 h. It can be seen that the change in pressure slightly affects the maximum amount of the adsorbed ibuprofen. At the same time, with increasing temperature, the maximum amount of the adsorbed ibuprofen into silica aerogel noticeably increasing. This phenomenon is not typical for physical adsorption, the value of which should decrease with increasing temperature. The result can be due to some reasons. The first is associated with the solubility of ibuprofen in supercritical carbon dioxide, the second with the possible presence of various conformations of ibuprofen in a supercritical solution, and the third with different phase behavior of system “carbon dioxide-ibuprofen”. As shown in [26], the solubility of ibuprofen in supercritical carbon dioxide at pressures of 140–200 bar increases with an increasing in temperature. The adsorption limit value is an equilibrium value and, in particular, is determined by the concentration of the adsorbed substance in the surrounding solution. Thus, the higher the concentration of ibuprofen in supercritical carbon dioxide, the higher should be the maximum adsorption value and, therefore, the maximum amount of the adsorbed ibuprofen into aerogel.

However, this does not explain the corresponding increase in amount of the adsorbed ibuprofen at a pressure of 120 bar, since according to the literature it is shown that at a given pressure, the solubility of ibuprofen practically does not change. The conformational polymorphism of ibuprofen in supercritical carbon dioxide was studied in [27]. It was shown that at a temperature of 40–80 °C and a carbon dioxide density of 1.3 × ρ_c_, two ibuprofen conformations (conformation I and conformation II) can exist, which can lead to the formation of two various crystalline structures of ibuprofen after the supercritical adsorption during precipitation at depressurization [28]. Moreover, with increasing temperature, there is a maximum occurrence probability of conformation I at a temperature of 333 K. Conformation I is more stable, which can cause an increase in the limiting value of adsorption with an increase in temperature from 313 to 333 K. 

**Figure 3:**
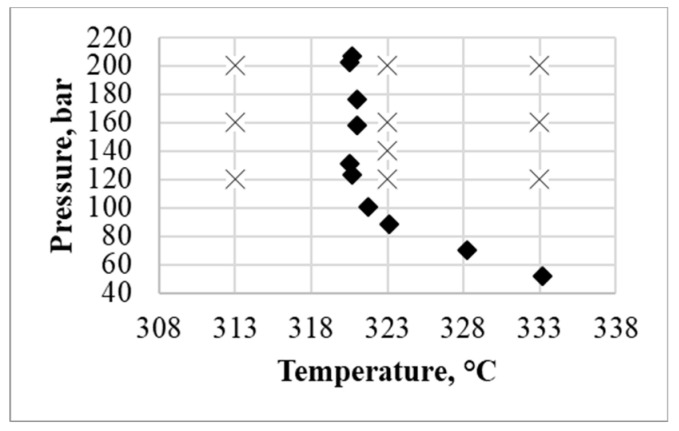
Phase behavior for carbon dioxide and RS-ibuprofen system, p-T projection of solid-liquid-gas line [29] (♦), and parameters of experimental investigation (×).

For understanding phase behavior of the studied two-component system p-T projection of solid-liquid-gas line for carbon dioxide and RS-ibuprofen system is presented in Figure 3 together with parameters of supercritical adsorption experiments.

As can be seen from the above data, the ibuprofen phase is in the liquid state on the right side of the solid-liquid-gas line, and in the solid state on its left side. Therefore, ibuprofen is in the solid state only at the 313 K and at other supercritical adsorption temperatures it is in liquid state. This may cause a change in the adsorption dependence on the solubility of ibuprofen at a temperature of 313 K. In any case, adsorption cannot be considered and predicted simultaneously at parameters that correspond to both the liquid and solid state of ibuprofen. Therefore, further, to calculate the coefficients of the model and its verification, we will use the data for which the loading of the final material is higher. For model coefficients calculation experimental data at temperature of 323 and 333 K and at pressure 120, 160, and 200 bar will used. Furthermore, for model verification data at temperature 323 K and pressure 140 bar will be used.

Finally, it can be concluded that supercritical adsorption of active substances into aerogels is a complex process that involves many phenomena. For a fundamental understanding of all these phenomena and the identification of basic patterns, additional research is needed.

## 5. Mathematical Model and the Results of Calculation

### 5.1. Mathematical Model

Mathematical model is based on provisions of continuum mechanics and represents a system of balance equations that include mass, momentum, and energy conservation equations for a homogeneous two-component system. Resulting system of differential equations is solved using numerical methods within a particular geometry. The following assumptions are made:

(1) The flow of two-component homogeneous system ”ibuprofen-supercritical fluid”, that is a viscous compressible fluid, is considered; the equipment works in periodic mode;

(2) The system consists of two calculation areas-free volume of the reactor and the volume of the porous body (aerogel); a surface that connects these areas is a velocity shear boundary;

(3) Mass transfer in a porous body is described by Fick’s diffusion equation without convective transport; in the porous body the adsorption takes place, which obeys the Langmuir theory;

(4) The temperature of the reactor wall is considered to be constant.

Model equations in the free volume of the reactor (calculation area *Θ*):

(1) $$\{\begin{array}{c} \begin{array}{c} \frac{\partial \left(\rho Y_{1}\right)}{\partial t}+𝛻\left(\rho \vec{v}Y_{1}\right)=𝛻\left(\rho D_{\Theta }𝛻Y_{1}\right)\\ \frac{\partial (\rho Y_{2})}{\partial t}+𝛻\left(\rho \vec{v}Y_{2}\right)=𝛻\left(\rho D_{\Theta }𝛻Y_{2}\right)\\ \frac{\partial (\rho \vec{v})}{\partial t}+𝛻\left(\rho \vec{v}\vec{v}\right)=-𝛻p+𝛻\left(\tau^{kl}\right)+\rho \vec{g}\\ \frac{\partial \left(\rho E\right)}{\partial t}+𝛻\left(\vec{v}\left(\rho E+p\right)\right)=𝛻\left(\lambda 𝛻T\right) \end{array} \end{array}$$

Model equations within the porous body (calculation area *Ω*):

(2) $$\{\begin{array}{c} \frac{\partial \left(\rho Y_{1}\right)}{\partial t}=𝛻\left(\rho D_{\Omega }𝛻Y_{1}\right)\\ \frac{\partial \left(\rho Y_{2}\right)}{\partial t}=𝛻\left(\rho D_{\Omega }𝛻Y_{2}\right)+\rho S_{q}\\ \frac{\partial (\rho E)}{\partial t}=𝛻\left(\lambda 𝛻T\right) \end{array}$$

the following initial and boundary conditions were set:

$$\begin{array}{c} \vec{v}{\left(x,y,z,t\right)}_{t=0}=0\\ T{\left(x,y,z,t\right)}_{t=0}=T_{init}\\ \forall x,y,z\in \Theta \;Y_{2}{\left(x,y,z,t\right)}_{t=0}=0\\ \forall x,y,z\in \Omega \;Y_{2}{\left(x,y,z,t\right)}_{t=0}=0\\ \forall x,y,z\in \Omega \;q{\left(x,y,z,t\right)}_{t=0}=0\\ \vec{v}\left(x_{s},\;y_{s},\;z_{s},t\right)=0\\ \vec{v}\left(x_{w},\;y_{w},\;z_{w},t\right)=0\\ \vec{v}{\left(x_{b},\;y_{b},\;z_{b},t\right)}_{\forall x_{b},\;y_{b},\;z_{b}\in \Theta }=0\\ T\left(x_{w},\;y_{w},\;z_{w},t\right)=T_{w}\\ 𝛻T{\left(x_{b},\;y_{b},\;z_{b},t\right)}_{\forall x_{b},\;y_{b},\;z_{b}\in \Theta }=𝛻T{\left(x_{b},\;y_{b},\;z_{b},t\right)}_{\forall x_{b},\;y_{b},\;z_{b}\in \Omega }\\ Y_{2}\left(x_{s},\;y_{s},\;z_{s},t\right)=Y_{s}\\ 𝛻Y_{2}\left(x_{w},\;y_{w},\;z_{w},t\right)=0\\ Y_{2}{\left(x_{b},\;y_{b},\;z_{b},t\right)}_{\forall x_{b},\;y_{b},\;z_{b}\in \Theta }=Y_{2}{\left(x_{b},\;y_{b},\;z_{b},t\right)}_{\forall x_{b},\;y_{b},\;z_{b}\in \Omega } \end{array}$$

where *ρ*-mixture density, kg/m^3^; $\vec{v}$-mixture velocity vector, m/s; T-mixture temperature, K; *p*-pressure, Pa; *Y*_1_-mass fraction of carbon dioxide kg/kg; *Y*_2_-mass fraction of active substance (*Y*_2_* = 1 − Y*_1_**), kg/kg; $\vec{g}$-gravitational acceleration, m/s^2^; *D_Θ,_ D_Ω_*-molecular diffusion coefficients (in the free volume of the reactor) and effective diffusion (within the porous body), respectively, m^2^/s; *λ*-heat-conductivity coefficient, W/(m∙K); *E*-total energy, J/kg; *S_q_*-source of mass during the adsorption; and index *w* corresponds to the values on the wall of the reactors, *b*-values on the border between the calculation areas, and *s*-values at the boundary of the source of mass of the active substance.

Additional correlations:

$$S_{q}=-KY_{2}\left(q_{\infty }-q\right)$$

$$\tau^{kl}=\mu \left[\left(𝛻\vec{v}+𝛻{\vec{v}}^{T}\right)-\frac{2}{3}𝛻\cdot \vec{v}I\right]$$

where *Y_1Ω_*-mass fraction of carbon dioxide at the border in the calculation area *Ω*, kg/kg_mix_; *K*-adsorption rate coefficient, kg_mix_/kg∙s; *q*-adsorption value relative to mass of mixture, kg/kg_mix_; *q_∞_*-adsorption limit value relative to mass of mixture, kg/kg_mix_; µ-viscosity, Pa∙s; and I-unit tensor, the right component of the equation characterizes the effect of volume expansion.

Peng–Robinson equation of state is used for density calculation [30]. Physico-chemical properties of the mixture are taken equal to the properties of carbon dioxide. 

The adsorption rate constant is calculated on the basis of the molecular-kinetic theory according to the following equation:

(3) $$K=\frac{pSM_{a}{}^{2}}{q_{\infty }M_{\mathrm{mix}}\sqrt{2\pi M_{a}RT}}$$

where *S*-specific surface area of the aerogel, m^2^/g; *M_a_*-molar mass of active substance, g/mol; and *M_mix_*-molar mass of the mixture (taken equal to the molar mass of the solvent), g/mol.

The adsorption limit characterizes the amount of active substance that is in the state associated with the inner surface of the aerogel, adsorbed upon reaching equilibrium. The maximum amount of the adsorbed active substance is a value that is determined from the experimental data of the kinetics of the process. It reflects the total amount of active substance that is included in the aerogel upon reaching equilibrium. It should be noted that inside the aerogel, during the process of supercritical adsorption, part of the active substance is dissolved in supercritical carbon dioxide, which is located inside the pores, and the other part is in an adsorbed state on the inner surface of the aerogel.

Thus, to calculate the adsorption limit, the following equation is used:

(4) $$q_{\infty }=\frac{A_{\infty }\rho_{aэp}}{\left(100-A_{\infty }\right)\rho \varepsilon }-Y_{p}$$

where *ρ_aэp_*-aerogel density, kg/m^3^; *ρ*-carbon dioxide density with supercritical adsorption process parameters, kg/m^3^; *A*_∞_-maximum amount of absorbed active substance into the aerogel, %mass; Y_P_-solubility of the active substance with parameters of the supercritical adsorption process, kg/kg; and ε-aerogel porosity. 

The equations of the model were solved using the Ansys Fluent 17.0 software (ANSYS, Canonsburg, PA, USA).

### 5.2. Results of Supercritical Adsorption Modeling

**Figure 4:**
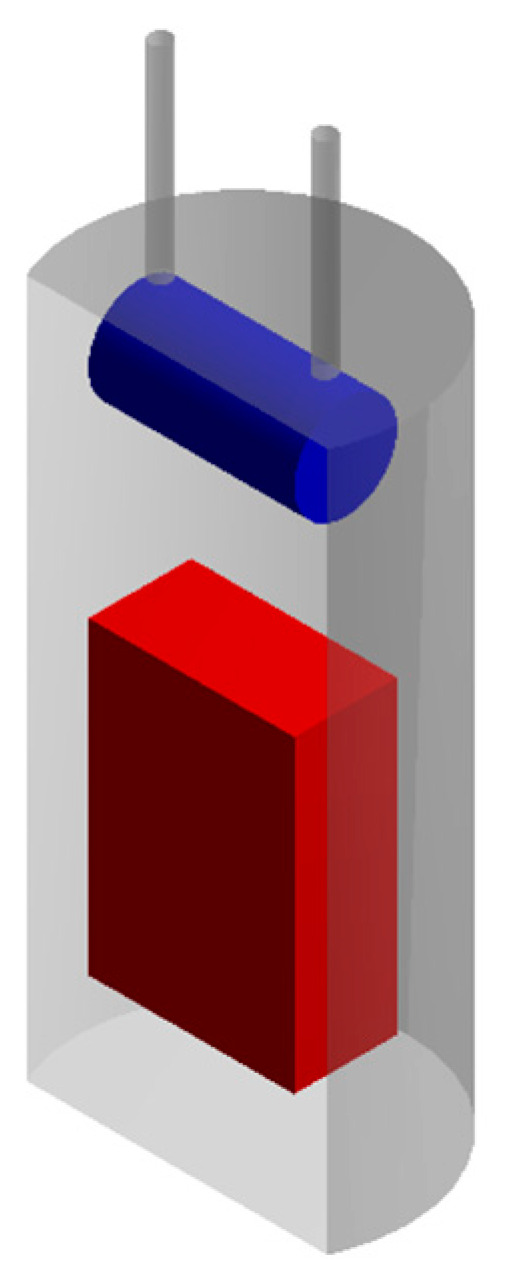
Geometry of the reactor for supercritical adsorption.

To describe the process of supercritical adsorption, the reactor’s geometry was constructed, which is shown in Figure 4. In the lower part of the reactor there is a cylinder representing a porous body (aerogel), in the upper part there is a parallelepiped characterizing an envelope with a sample of the active substance. The calculation of the model equations was carried out at parameters corresponding to experimental studies. The physicochemical properties of supercritical carbon dioxide [30], the solubility of ibuprofen in supercritical carbon dioxide at various process parameters was determined from reference data [26,31,32,33,34].

To determine the molecular diffusion coefficient for the free volume of the reactor, the He and Yu equation was used [35]. The dependence of the effective diffusion coefficient in the volume of the porous body on temperature, pressure, and the temperature dependence of the maximum amount of adsorbed active substance into the aerogel were determined using experimental data. It is assumed that these dependencies have the following form:

(5) $$D_{\Omega }\left(T,p\right)=\left(a_{1}\cdot p+a_{2}\right)e^{-\frac{a_{3}\cdot P+a_{4}}{T}}$$

(6) $$A_{\infty }=\left(b_{1}\cdot T+b_{2}\right)\cdot 100$$

where *a_1_, a_2_, a_3_, a_4_, b_1_,* and *b_2_*-empirical coefficients.

To determine the values of empirical coefficients, six experimental curves of the kinetics of supercritical adsorption were used (Figure 2) at pressures of 120, 160, and 200 bar and temperatures 323, 333 K. The calculation was carried out according to the proposed model of the kinetics of the process of supercritical adsorption. To find the minimum discrepancy between the calculated and experimental data when varying the effective diffusion coefficient, the golden section method was used. Table 1 presents the obtained values of empirical coefficients.

**Figure 5:**
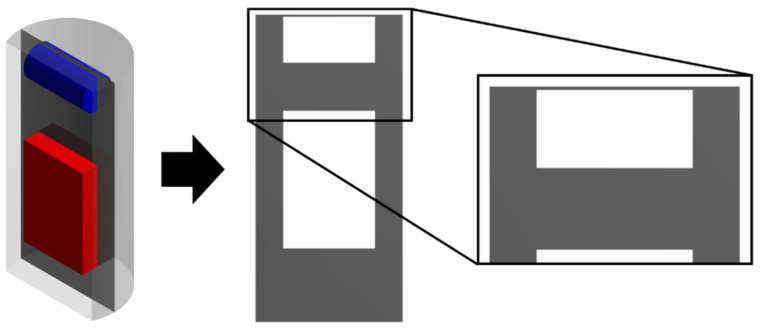
Cross-section surface for free volume of the reactor (Θ).

**Figure 6:**
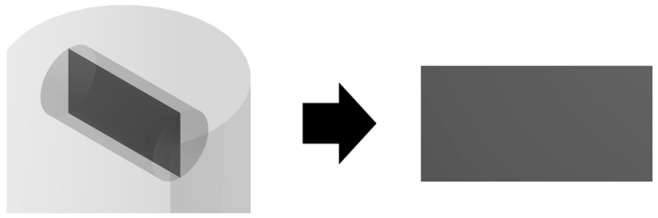
Cross-section surface for porous body (Ω).

**Figure 7:**
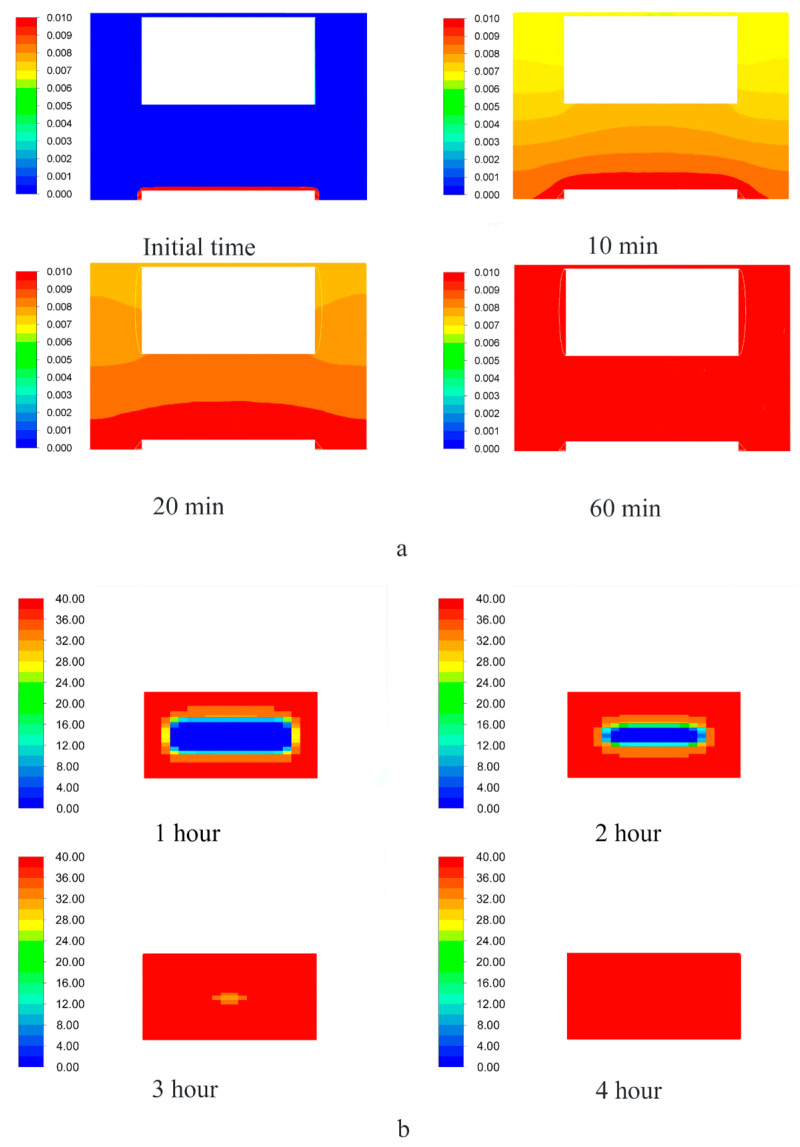
The distribution of the content of the active substance in the free volume of the reactor (**a**) and total amount of adsorbed active substance inside the porous body (aerogel) (**b**) at P = 140 bar and T = 323 K.

The results of each calculation of the equations of the kinetics model of the supercritical adsorption process are the fields of velocity, pressure, composition, and other parameters of the system inside the apparatus and their change in time. For greater clarity, the data obtained are displayed on secant planes: Secant plane for the free volume of the reactor (Figure 5) and the volume of the porous body (Figure 6). For example, Figure 6 presents the results of one of the calculations: The distribution of the active substance content at each point of the reactor (Figure 7a), the amount of active substance inside the porous body (Figure 7b). Figure 7a shows the saturation of supercritical carbon dioxide with an active substance (blue—the minimum content, red—the maximum, which corresponds to the solubility under these conditions). Figure 7b shows the change in the content of the active substance at each point of the porous body from the minimum (aerogel without active substance-blue) to maximum (red), which corresponds to the amount of the adsorbed active substance.

**Figure 8:**
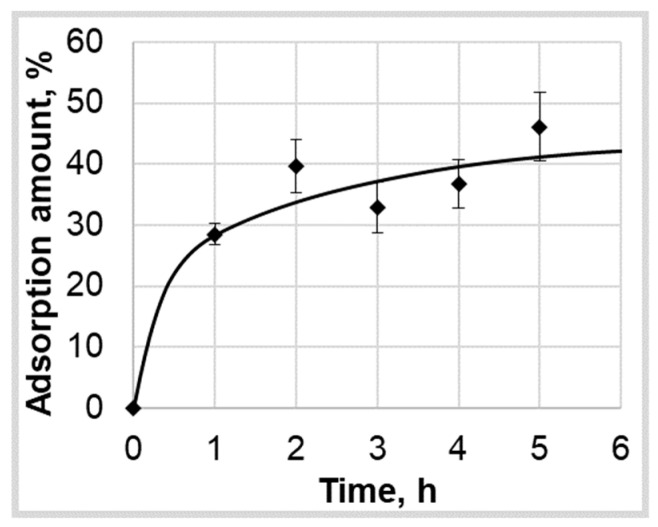
Experimental (◆) and calculated (**−**) data of ibuprofen supercritical adsorption into silica aerogel kinetics at pressure 140 bar and temperature 323 K.

To determine the calculated total amount of adsorbed active substance into the aerogel using Ansys Fluent instruments within the porous body (calculated region Ω), the mass of the active substance in the pore volume and the mass of the active substance in the adsorbed state were determined. To verify the obtained equations, as well as to confirm the adequacy of the model, the ibuprofen adsorption was calculated at a pressure of 140 bar and a temperature of 323 K. The corresponding experimental data were not used to determine the diffusion coefficient and limiting adsorption. Figure 8 shows the calculated kinetics curves of supercritical adsorption and the corresponding experimental data.

It was found that the mathematical model adequately describes the experimental data with a relative error 8.9%. Thus, the approach presented in the work for calculating the supercritical adsorption process can be applied to any active substances and aerogels of various nature. The only requirement for solving such problems is the availability of relevant experimental data for calculating the necessary model coefficients to determine the dependence of the diffusion coefficient and the maximum amount of adsorbed active substance into the aerogel on the process parameters. The mathematical model allows study of the influence of hydrodynamics, heat, and mass transfer on the course of the process of supercritical adsorption at each point of the studied region in devices of different geometries.

The approach presented in the work can also be applied to calculate the process of obtaining various materials based on aerogels. Each new pair of active substance-aerogel requires an experimental study of the kinetics of the supercritical adsorption process to determine all the necessary coefficients. Such an experiment does not require the use of industrial equipment; it is relatively cheap.

## 6. Conclusions

An experimental study of the kinetics of supercritical adsorption was carried out on the example of obtaining the composition ”ibuprofen-silica aerogel“ at various temperatures and pressures. It was shown that the process of supercritical adsorption of active substances includes many phenomena that are associated with both the special properties of carbon dioxide and the molecular structure of the active substance and aerogel. To implement this study, an equipment of own design was developed.

A mathematical model of the supercritical adsorption process based on the provisions of continuum mechanics is proposed. The calculation of model equations is carried out using the Ansys Fluent software package. After a series of calculations and comparing them with experimental data, the dependence of the effective diffusion coefficient in the region of the porous body (aerogel) on temperature and pressure is obtained. Using the data obtained, the model is verified. 

The mathematical model proposed in this work can be used to calculate the process of supercritical adsorption of various active substances into various porous bodies. Using the proposed approach will reduce resource and energy costs of the supercritical adsorption process. In addition, its use will allow the development of new equipment, including those on an industrial scale.

## Figures and Tables

**Table 1:** Empirical coefficients for calculating the diffusion coefficient and maximum amount of adsorbed active substance into the aerogel.

Empirical Coefficient	Unit Measure	Value
a_1_	m^3^∙s/kg	0.0125
a_2_	m^2^/s	−0.74
a_3_	K/bar	7.50
a_4_	K	4800
b_1_	kg/(kg∙K)	0.007
b_2_	kg/kg	−1.802
